# Predictors of Arrhythmia Recurrence After Cryoballoon Ablation for Persistent Atrial Fibrillation: A Sub‐Analysis of the Cryo Global Registry

**DOI:** 10.1111/jce.16571

**Published:** 2025-01-27

**Authors:** K. R. Julian Chun, Christoph Stellbrink, Masato Murakami, Christian Drephal, Il‐Young Oh, Kelly A. van Bragt, Daniel Becker, Sylvain Anselme, Derick Todd, Krzysztof Kaczmarek, Junichi Nitta

**Affiliations:** ^1^ Cardioangiologisches Centrum Bethanien Frankfurt Germany; ^2^ Universitat Bielefeld Medizinische Fakultat OWL Bielefeld Germany; ^3^ Shonan Kamakura Sogo Byoin Nosocchu Shinryoka Kamakura Japan; ^4^ Sana Klinikum Lichtenberg Berlin Germany; ^5^ Seoul National University Bundang Hospital Seongnam South Korea; ^6^ Medtronic Minneapolis MN United States; ^7^ Medtronic France SAS Paris France; ^8^ Liverpool Heart and Chest Hospital NHS Foundation Trust Liverpool UK; ^9^ Uniwersytet Medyczny w Lodzi Klinika Kardiologii Łódź Poland; ^10^ Sakakibara Kinen Byoin Fuchu Japan

**Keywords:** catheter ablation, cryoballoon, persistent atrial fibrillation, predictors, registry

## Abstract

**Introduction:**

Freedom from recurrences of atrial tachyarrhythmia (ATA) is suboptimal after pulmonary vein isolation (PVI) in patients with persistent atrial fibrillation (PsAF). This sub‐analysis from the Cryo Global Registry sought to investigate predictors of ablation success after PVI using cryoballoon ablation (CBA) for PsAF.

**Methods and Results:**

ATA recurrence was defined as ≥ 30 s recurrence of atrial fibrillation, atrial flutter or atrial tachycardia after a 90‐day blanking period and through 12‐months. Univariate and multivariable Cox regression analysis (with ATA recurrence as an endpoint) was performed to identify CBA responders. PsAF patients (*N* = 882) were on average 63.9 ± 11.3 years old (69.2% male), and freedom from ATA recurrence was 79.7% (76.8%–82.2%). Longer elapsed time from PsAF diagnosis to ablation (hazard ratio [HR] 1.07 (95% confidence interval [CI]: 1.03–1.11); *p* = 0.002) and a larger number of previously failed antiarrhythmic drugs (HR 1.39 (95% CI: 1.13–1.70); *p* < 0.002) were shown to be independent predictors of ATA recurrence in a multivariate model which included 703 evaluated patients.

**Conclusion:**

These real‐world results provide important insights to guide referral of PsAF patients, including the benefits of earlier treatment via CBA.

## 1 Introduction

Catheter ablation using the cryoballoon is a well‐established method to improve symptom burden and quality of life in patients with atrial fibrillation (AF). Yet, freedom from atrial tachyarrhythmia (ATA) recurrences is suboptimal in patients with persistent AF (PsAF) [1, 2]. This sub‐analysis of the Cryo Global Registry (NCT02752737) aimed to identify which patient baseline characteristics predict ATA recurrences after cryoballoon ablation in a large patient cohortwith PsAF to potentially inform better treatment.

## 2 Methods

All patients (≥ 18 years) undergoing cryoballoon ablation (CBA) with arctic front (Advance) (Medtronic Inc) were eligible for enrollment. PsAF patients (AF episodes ≥ 7 days and ≤ 12 months) enrolled between June 2016 and July 2021 in 31 countries with 12‐month follow‐up data available, were included. Patients with a prior atrial ablation were excluded. Study conduct was described previously [3]. In brief, the study was approved by independent ethics committees at each participating center, and all patients provided written informed consent before participating. Procedures were conducted according to standard‐of‐care. A 23‐ or 28‐mm cryoballoon was introduced into the left atrium via transseptal puncture using a dedicated, 15‐F steerable sheath (FlexCath (Advance) Steerable Sheath; Medtronic Inc). The cryoballoon was subsequently delivered to the targeted pulmonary vein (PV) with a J‐tip guidewire or dedicated circular mapping catheter (Achieve (Advance), Medtronic Inc). Operators were required to confirm PV isolation (PVI) by entrance and/or exit block testing after the ablation. Patients were followed up via telephone and/or in‐person office visits according to the centers' standard‐of‐care protocol.

### 2.1 Statistical Analysis

The primary endpoint was freedom from ATA recurrence, defined as freedom from a ≥ 30 s recurrence of AF, atrial flutter, or atrial tachycardia following a 90‐day blanking period. Incidences of recurrences were analyzed as time‐to‐first‐event. The Kaplan–Meier (KM) method was used to estimate time‐to‐incidence rates, with 12‐month freedom from recurrence rates being reported. Group comparisons, patients with versus without ATA recurrence, were performed by a priori potential predictors. Differences in predictors between groups were analyzed using Wilcoxon Tests and Chi‐square or Fisher's Exact Tests for continuous and categorical variables, respectively. Furthermore, univariate cox regression was performed for each predictor to report the hazard ratio (HR) with 95% confidence intervals (CIs). For the multivariate Cox regression model a stepwise selection process for the predictors included, was used with entry level *p* = 0.20 and stay criteria *p* = 0.20 with AT/AF/AFL as the dependent variable. Excluded from the model were variables missing in more than 20% of the subjects.

## 3 Results

**Figure 1:**
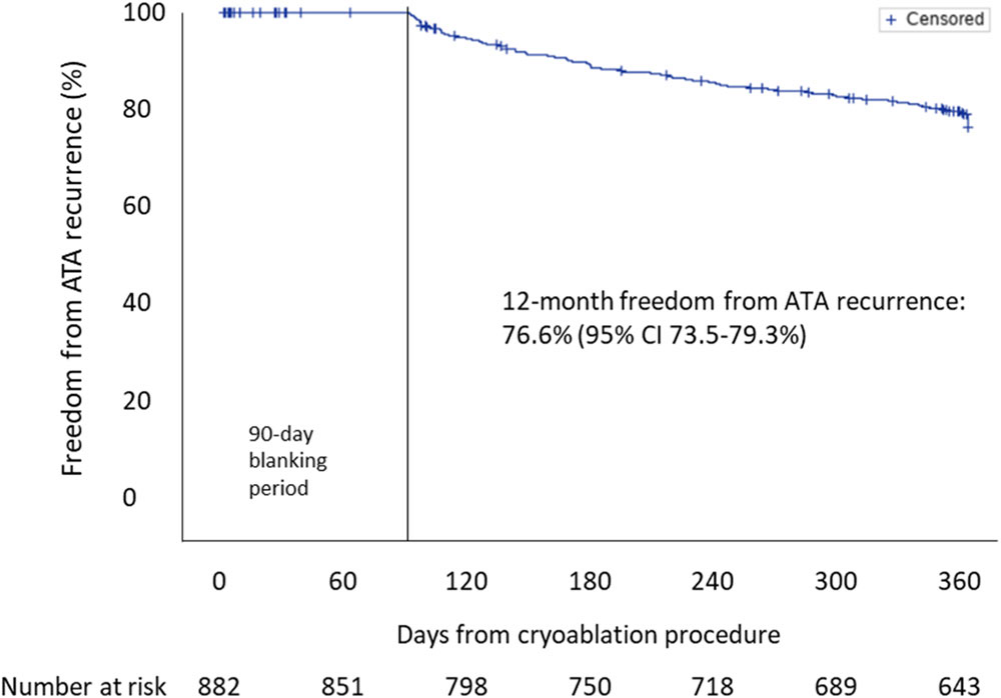
Freedom from atrial tachyarrhythmia (ATA) recurrence at 12 months in persistent atrial fibrillation (AF) patients undergoing cryoballoon ablation.

**Figure 2:**
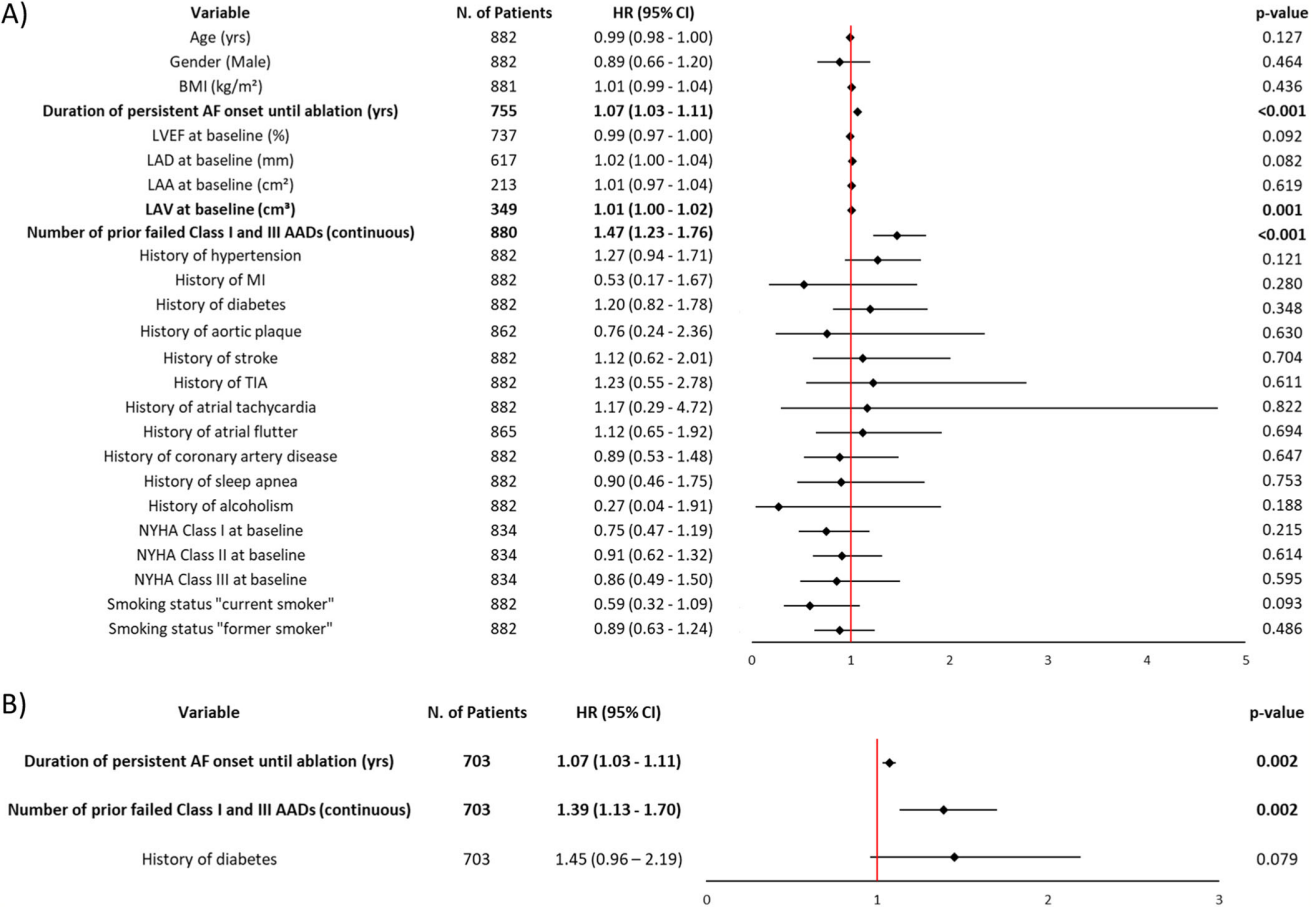
Univariate (A) and multivariate (B) Cox regression analysis. AADs, arrhythmic drugs; AF, atrial fibrillation; BMI, body mass index; CI, confidence interval; HR, hazard ratio; NYHA, New York Heart Association.

Amongst 882 patients analyzed, 83 (9.4%) patients received a repeat ablation, and 305 (37.8%) patients were on arrhythmic drugs (AADs) at 12 months FU. The majority of PsAF patients received PVI only (79.5%), using the study catheter during the initial ablation. Lesions beyond PVI (PVI + ) were done in 181 (20.5%) patients with CTI in 116 (13.2%) patients and lnon‐CTI lesions in 96 (10.9%) patients. The majority of these patients, 65 (7.4%) received left‐sided rooflines. Freedom from ATA recurrence at 12 months in patients with PVI+ was 86.9% (95% CI: 80.7–91.1%) and in all patients 76.6% (95% CI: 73.5–79.3, Figure 1). Compared to all patients without ATA recurrence, those with recurrent ATA: (1) had longer elapsed time from PsAF diagnosis to index CBA procedure (0.9 (0.3–2.4] vs. 0.5 (0.2–1.4) years, *p* < 0.001); (2) had larger left atrial diameter (44.4 ± 5.8 vs. 43.4 ± 7.5 mm, *p* = 0.037); and (3) failed more AADs before ablation (0.8 ± 0.8 vs. 0.6 ± 0.7 AAD failures, *p* < 0.001; Table 1). There was no difference in serious procedure‐related adverse events between patients with ATA (5.1%) and without ATA (3.1%, *p* = 0.18). The univariate Cox regression analysis including all 882 patients is presented in Figure 2A. Patients with longer PsAF diagnosis‐to‐ablation time (HR = 1.07; 95% CI: 1.03–1.11), a higher number of prior failed AADs (HR = 1.47; 95% CI: 1.23–1.76), and larger left atrial volume (HR = 1.01; 95% CI: 1.00–1.02) at baseline had a significantly higher risk of ATA recurrence post CBA. In a multivariate model including 703 patients (Figure 2B), PsAF diagnosis‐to‐ablation time (HR = 1.07; 95% CI: 1.03–1.11; *p* = 0.002) and number of prior failed AADs (HR = 1.39; 95% CI: 1.13–1.70; *p* < 0.002) were identified as independent predictors of ATA recurrence. Measurements of left atrial area, left atrial volume, left ventricular ejection fraction, and left atrial diameter were not included in the multivariate model as they were missing in more than 20% of the patients.

**Table 1:** Patient baseline characteristics.

Baseline patient characteristics	Without ATA recurrence (*N* = 687)	With ATA recurrence (*N* = 195)	*p*‐Value
Age (years)	64.2 ± 11.1	62.7 ± 11.9	0.184**
Body mass index (kg/m^2^)	27.7 ± 5.5	28.1 ± 5.4	0.430**
Gender (male)	69.9% (480/687)	66.7% (130/195)	0.393*
Diagnosed with persistent AF (years)	0.5 [0.2–1.4]	0.9 [0.3–2.4]	**< 0.001** **
Left atrial diameter (mm)	43.4 ± 7.5	44.4 ± 5.8	**0.037** **
Left atrial area (cm^2^)	26.0 ± 7.7	26.6 ± 6.3	0.228**
Left atrial volume (cm^3^)	76.2 ± 27.5	87.8 ± 31.9	**0.004** **
Left ventricular ejection fraction (%)	56.2 ± 10.6	54.7 ± 11.2	0.134**
Number of failed antiarrhythmic drugs (Class I or III)	0.6 ± 0.7	0.8 ± 0.8	**< 0.001** **
History of atrial tachycardia	0.9% (6/687)	1.0% (2/195)	0.843*
History of atrial flutter	6.2% (42/673)	7.3% (14/192)	0.602*
NYHA at baseline			
Subject does not have heart failure	57.7% (374/648)	62.4% (116/186)	0.647*
I	14.4% (93/648)	11.3% (21/186)	
II	19.9% (129/648)	18.8% (35/186)	
III	8.0% (52/648)	7.5% (14/186)	
Hypertension	60.6% (416/687)	67.7% (132/195)	0.070*
Prior myocardial infarction	2.9% (20/687)	1.5% (3/195)	0.288*
Prior stroke	4.9% (34/687)	6.2% (12/195)	0.504*
Prior transient ischemic attack	2.5% (17/687)	3.1% (6/195)	0.641*
History of coronary artery disease	9.3% (64/687)	8.2% (16/195)	0.634*
History of diabetes	13.4% (92/687)	15.4% (30/195)	0.477*
History of sleep apnea	5.2% (36/687)	4.6% (9/195)	0.726*
History of alcoholism	2.0% (14/687)	0.5% (1/195)	0.146*
Smoking status			
None	66.7% (458/687)	71.8% (140/195)	0.222*
Current	9.2% (63/687)	5.6% (11/195)	
Former	24.2% (166/687)	22.6% (44/195)	

*Note:* Data are presented as mean ± standard deviation or % subjects (N subjects/total subjects).

Abbreviations: AF, atrial fibrillation; ATA, atrial tachyarrhythmia; NYHA, New York Heart Association.

* Chi‐square test.

** Wilcoxon test.

## 4 Discussion

Main findings form this real‐world global analysis of PsAF patients treated by CBA included the following: (1) 12‐month freedom from ATA recurrence was 76.6% and (2) CBA responders had a shorter time from PsAF diagnosis to ablation and a lower number of prior failed AADs.

## 5 Predictors of Recurrence After CBA in Paroxysmal and Persistent AF

Two meta‐analyses describe predictors of recurrence after CBA in patients with AF [4, 5]. Both meta‐analyses found that duration of AF history (diagnosis‐to‐ablation time), left atrial diameter, and PsAF were baseline predictors of AF recurrence. Song and colleagues highlight that the studies used had predominantly paroxysmal AF (PAF, 74%), and therefore, it is unclear whether the results can be extrapolated to all PsAF patients. In a recent analysis by Stabile and colleagues, predictors of recurrence were compared between PAF and PsAF patients undergoing CB‐PVI within the 1STOP registry [6]. Female sex and time from first symptomatic AF episode were shown to be significant predictors for AF recurrence in PAF. In patients with PsAF, time from first PsAF (episode) and AAD usage were found to be independent predictor of AF recurrence, similar to this analysis.

## 6 Timing of Ablation in Persistent AF

AF is a self‐perpetuating arrhythmia inducing atrial remodeling and promoting AF disease progression [7]. The current findings suggest that the time spend in PsAF is an important factor for the prediction of procedural failure because AF can potentiate more deleterious AF. The two identified predictors of recurrence in PsAF patients are specifically of interest because they can be influenced by early referral of patients for CBA. This is in alignment with current AF guidelines that emphasize the importance of early and continued AF management focused on maintaining sinus rhythm and minimizing AF burden [8]. Takamiya et al. [9] showed that longer PsAF diagnosis‐to‐ablation time predicted non‐PV AF/ATA triggers that were associated with ATA recurrence. Furthermore, De Greef et al. [10] identified time‐to‐ablation as an important predictor of AF recurrence in PAF and PsAF patients, but earlier timing of ablation seemed even more paramount in patients with PsAF than PAF to improve outcome [10].

### 6.1 Limitations

This is an Ad Hoc analysis of the Cryo Global Registry, and consequently, was neither powered nor was analysis predefined. Also, due to the standard‐of‐care nature of this registry, left atrial dimension where only available in a small subset of patients (*N* = 349) and therefore not included in the multivariate model. In addition, the standard‐of‐care monitoring within the registry framework may have resulted in underreporting of asymptomatic recurrences and introduction of bias due to different monitoring strategies. Furthermore, time from PsAF diagnosis to ablation is only a surrogate for time spent in AF and AF burden reduction would be clinically more relevant than time to first AF recurrence.

## 7 Conclusion

Longer PsAF diagnosis‐to‐ablation time and a higher number of previously failed AADs were shown to be independent predictors of ATA recurrence in a real‐world population of PsAF patients undergoing CBA. This analysis provides important insights to guide early referral and treatment of PsAF patients.

## Disclosures

K. R. Julian Chun, Christoph Stellbrink, Masato Murakami, Junichi Nitta, and Derick Todd received speaker fees from Medtronic. K. R. Julian Chun and Christoph Stellbrink received research grants from Medtronic. Krzysztof Kaczmarek received lecturer and consultant fees from Medtronic. Kelly A. van Bragt, Daniel Becker and Sylvain Anselme are employees of Medtronic. The other authors have nothing to declare.

## Data Availability

The data that support the findings of this study are not available on request due to privacy restrictions.
